# The Pathway for New Cancer Drug Access in Canada

**DOI:** 10.3390/curroncol29020041

**Published:** 2022-01-21

**Authors:** Joanna Gotfrit, William Dempster, Johanne Chambers, Paul Wheatley-Price

**Affiliations:** 1 Division of Medical Oncology, Department of Medicine, The Ottawa Hospital and University of Ottawa, Ottawa, ON K1H 8L6, Canada; jgotfrit@toh.ca; 2 Ottawa Hospital Research Institute, Ottawa, ON K1Y 4E9, Canada; 3 3Sixty Public Affairs, Ottawa, ON K2P 1P1, Canada; wdempster@3sixtypublicaffairs.com (W.D.); jchambers@3sixtypublicaffairs.com (J.C.)

**Keywords:** cancer drugs, health technology assessment, funding, access

## Abstract

Cancer treatment has evolved significantly over the past decade with the emergence of a multitude of new treatments across cancer types. Alongside the pace of drug discovery, the cost of cancer drugs has also increased. In the face of this growth in development and spending, it is crucial to have an understanding of the processes and pressures new drugs navigate to get to the market in Canada. This paper is a review of the complex, multi-step regulatory and funding process undertaken by cancer drugs in Canada. It reviews the role of Health Canada, the Patented Medicine Prices Review Board, the Health Technology Assessment, the pan-Canadian Pharmaceutical Alliance, and finally, the provincial, territorial, and federal payers. Recent developments are highlighted. Strategies to minimize duplication of effort, improve timeliness, and increase efficiency are explored. The cancer drug regulatory and funding process in Canada is complex, and an understanding of the current system and ongoing evolution is essential.

## 1. Introduction

Cancer treatment has evolved significantly over the past decade with the emergence of dozens of new treatments across cancer types. New intravenous therapies and new classes of agents have become the standard of care. Drug-antibody conjugates have emerged, such as Ado-trastuzumab emtansine, which has become established in breast cancer; monoclonal antibodies targeting angiogenesis, such as bevacizumab, are approved in colorectal cancer; and immunotherapy in the form of checkpoint inhibitors, such as pembrolizumab, nivolumab, cemiplimab, avelumab, durvalumab and atezolizumab, have revolutionized outcomes for thousands of cancer patients with tumours as diverse as lung cancer, melanoma, kidney cancer, and bladder cancer, to name a few. In addition, multiple new oral cancer drugs are now available as targeted therapies for multiple cancer types, perhaps most clearly in the identification and treatment of multiple subtypes of lung cancer defined by targetable driver mutations.

Alongside the pace of drug discovery, the cost of these drugs has also increased. The Patented Medicines Prices Review Board (PMPRB) released a report in 2020 describing the change in cost of cancer drugs over the prior decade [1]. This report describes a tripling of annual spending on cancer drugs to $3.9 billion in 2019, representing 14.6% of the drugs market compared to 7.1% in 2009.

In the face of this growth in development and spending, it is important to have an understanding of the processes and pressures new drugs navigate to get to the market in Canada, in order to make the system effective, equitable, affordable and sustainable. This paper outlines the multiple steps in Canada’s relatively complicated system and some of the changes that are occurring or are in discussion. We limit the manuscript to the description of new, patented, or single-source cancer drugs, rather than off-patent or generic medications.

### 1.1. The Process

**Figure 1:**
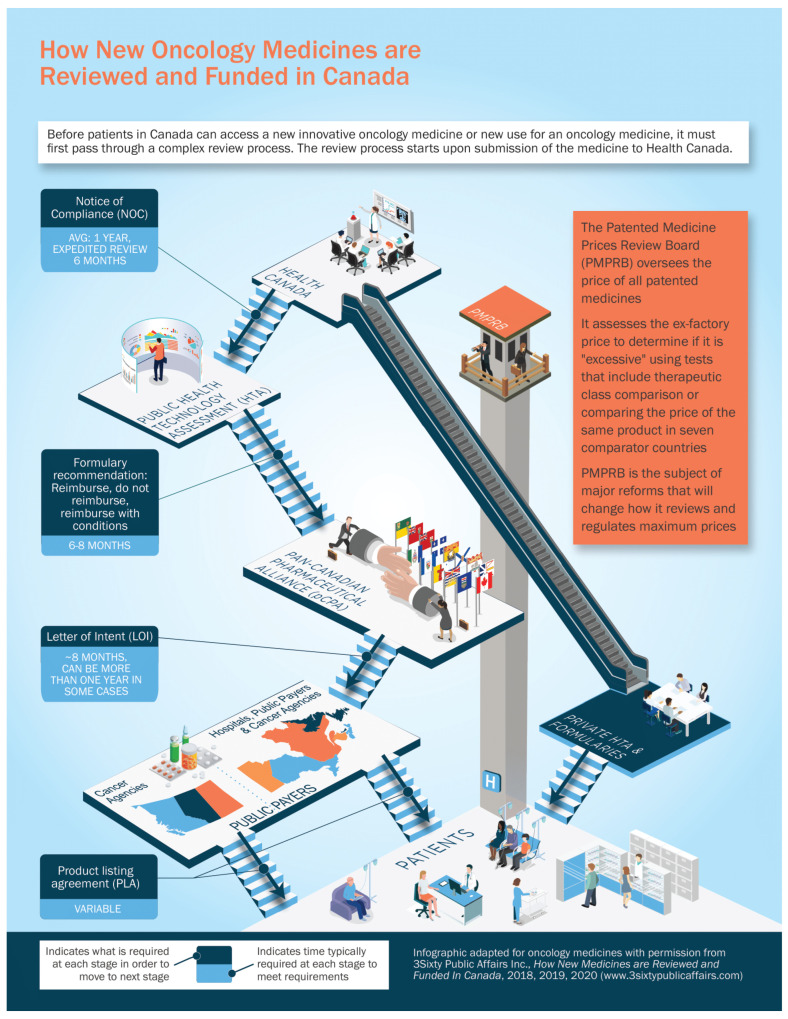
Summary of the cancer drug review and funding process in Canada. Reprinted with permission from Ref. [2].

For a new cancer drug to make it from a successful clinical trial to publicly reimbursed standard of care, there are multiple regulatory, evaluation, and funding steps. We will outline them initially very briefly here, and then will go into more detail on each step. See Figure 1 for a summary of the process.

Health Canada (HC): Acts as the federal regulator authorizing drugs to be sold in Canada when satisfied that they are safe, effective, and of high quality.

Patented Medicines Prices Review Board (PMPRB): Its role is to monitor and regulate the maximum (ceiling) prices of patented medicines sold in Canada.

Public Health Technology Assessment: For all provinces and territories except Quebec, the Canadian Agency for Drugs and Technologies in Health (CADTH) undertakes comparative clinical and economic reimbursement reviews. CADTH makes non-binding recommendations to public payers on whether new cancer drugs should be approved for public reimbursement. The Institut national d’excellence en santé et en services sociaux (INESSS) performs the health technology reviews for Quebec.

Pan-Canadian Pharmaceutical Alliance (pCPA): If recommended by CADTH/INESSS, the pCPA then determines whether Canadian public plans (all provinces, territories, and certain federal drug programs) wish to enter into confidential negotiations about how much they should pay for an HC approved cancer drug. If the plans indicate a willingness to negotiate, the pCPA office or one jurisdiction leads negotiations on behalf of all the public programs. Drugs rejected by the participating plans (i.e., pCPA or individual plans decline to participate in negotiations or negotiations do not result in agreement) are generally not eligible for public funding even though they have been approved by HC. Successful negotiations lead to the signing of a letter-of-intent (LOI) between the manufacturer and the public plans collectively. The LOI outlines common terms that are used as a basis for product listing agreements (PLAs) that are subsequently finalized with individual public plans.

Provinces and Territories: Finally, when a drug has been approved and a Canadian price agreed upon, each jurisdiction makes the final decision whether to add it to their own formulary of publicly funded cancer medications based on the pCPA-negotiated LOI terms. If the drug is funded, a PLA is completed, and the product is added to the jurisdiction’s formulary.

### 1.2. Health Canada

Without HC authorization, no new drug can be marketed in Canada by the sponsor. Clinicians may apply for access to unapproved products through the special access program (SAP); however, SAP medicines are not generally publicly reimbursed and are only approved for limited time periods and courses of treatment. 

The Health Products and Food Branch (HPFB) is the section of Health Canada that reviews a new drug seeking market access when a formal “New Drug Submission” is filed by the manufacturer (or sponsor). The HPFB reviews the safety, efficacy, and quality of the new drug based on the clinical trial and manufacturing information submitted. If the review is positive, concluding that the drug is safe, effective, and of high quality, determining that benefits outweigh risks and that risks can be mitigated, then HC grants a “Notice of Compliance” (NOC) and the medication is assigned a “Drug Identification Number” (DIN). The NOC and DIN then allow the sponsor to market the drug in Canada [3].

HC works to a timeline with a general commitment to making an adjudication within 300 days [4]. HPFB also has a “Priority Review Process” for new drugs that are intended to treat, prevent, or diagnose serious, life-threatening, or severely debilitating illnesses or conditions where (a) there is no existing drug on the Canadian market with the same profile or (b) where the new product represents a significant improvement in the benefit/risk profile over existing products. A priority review reduces the target timeframe for a decision to 180 days but does not alter the substantive elements of the review.

In certain cases where there are gaps in the clinical evidence, Health Canada can issue a Notice of Compliance with conditions (NOC/c) that can be granted to drugs in order to provide expedited access to life-saving treatments. Cancer drugs are often addressed through this pathway. To be eligible, drugs must represent a significant improvement in treatment in a therapeutic area with no alternative options. The “conditions” imposed by an NOC/c are generally commitments by the sponsor to undertake further studies to confirm the safety and efficacy of the drug. For example, the drug alectinib, which is used in the treatment of anaplastic lymphoma kinase positive non-small cell lung cancer (ALK+ NSCLC), was granted NOC/c in September 2016. Following additional clinical trial data, the conditions were met, and full NOC was granted in September 2018 [5]. 

Project Orbis is a recent international initiative involving the USA, Canada, Singapore, Australia, Brazil, Switzerland, and the United Kingdom with the goal of speeding cancer drugs through the regulatory system. Starting in May 2019, it began assessing drugs that are eligible for priority review or those for which an NOC/c is being considered. The selection and coordination of Project Orbis applications are initiated by the US Food and Drug Administration (FDA), but the sharing of reviews and exchange of information are allowing for more rapid approvals, such as the approval of lenvatinib and pembrolizumab for endometrial cancer in September 2019. Project Orbis has been shown to reduce the delay between marketing application submission and approval in participating countries, with several applications shown to obtain timelines similar to the FDA [6]. A concern in Canada is that the HC approval does not translate into funded access. Whereas FDA approval generally brings insurance coverage and Medicare/Medicaid funding in the US, and a positive HTA approval from the National Institute for Health and Care Excellence (NICE) in the United Kingdom ensures funding, a rapid NOC in Canada in no way speeds up the HTA or pCPA processes that still need to occur in this country. At best, in Canada, Project Orbis may allow private access to cancer medications sooner. At worst (given the limited clinical data available at Health Canada approval) it creates a backlog for later steps that would normally require Phase III clinical data to address requirements from Health Technology Assessment agencies that evaluate new medicines for comparative clinical effectiveness.

### 1.3. PMPRB

The Patented Medicine Prices Review Board (PMPRB), created in 1987, is an independent, quasi-judicial body operating at arm’s-length from the federal Minister of Health. Its role is to regulate the prices of patented medicines sold in Canada [7]. Effectively, it sets the ceiling price (the Maximum Average Potential Price) at which patented medicines can be sold in Canada and ensures compliance [8].

To help guide the determination of the ceiling price, the PMPRB regulatory process includes a scientific review (which assesses the therapeutic benefit of the drug) and ongoing assessment to ensure the drug price is not excessive based upon comparison with other medicines of the same class, prices in other countries, changes in the consumer price index, and other factors [9,10]. Price review records are publicly available [11]. For example, when the drug bevacizumab (^®^Avastin, Genentech, Inc., South San Francisco, CA, USA) was reviewed, its Canadian price was found to be within the guidelines because it did not exceed the median price of the drug in other countries [12]. 

If the PMRPB finds that a drug price appears to exceed the guidelines, an investigation is triggered which may result in a voluntary compliance undertaking (VCU) whereby the patentee reduces the price and offsets excessive revenue through a payment to governments or other conditions. If a VCU is not forthcoming, the PMPRB can initiate and hold a public hearing to determine if the price is excessive and issue an order to compel the drug sponsor to reduce the price and remit excess revenues to the government [9]. It is important to note that drug manufacturers may experience substantial uncertainty around the possibility of PMPRB setting a lower price than anticipated, which may be perceived as a barrier to marketing a drug in Canada [13]. 

The PMPRB’s regulatory framework has experienced little change since 1987. According to the PMPRB, list prices of patented medicines in Canada are now among the highest in the world, and it now relies on outdated regulatory tools and information. As such, in 2019, the federal government amended the regulations governing the PMPRB to update its schedule of comparator countries, change its patentee reporting requirements (to secure access to “net” prices as a result of PLAs), and add new factors for the PMPRB to consider when determining whether a price is excessive, including market size and pharmacoeconomic value [14]. 

The PMPRB insists that these amendments, which have not yet been implemented by the federal government, would result in public, private, and out-of-pocket savings without significantly impacting medicine industry employment or investment in Canada. Nonetheless, the impending changes have sparked concerns of diminishing research investment and drug marketing in Canada [15]. The government has delayed the coming into force of the regulations several times and exempted COVID-19 therapies and vaccines from certain aspects of the reforms.

### 1.4. Health Technology Assessment

With HC approval, a drug can be sold in Canada but is not yet publicly reimbursed by public payers. Before the provinces and territories of Canada make a decision on whether they will publicly fund a cancer drug, the drug undergoes an HTA review. Between 2007 and 2010, these reviews (the Joint Oncology Drug Review, or JODR) were undertaken in Ontario on behalf of all the other provinces and territories (except Quebec) [16]. Subsequently, the pan-Canadian Oncology Drug Review (pCODR) took over and was later absorbed by CADTH. 

After receiving a submission, CADTH conducts an objective review of the clinical data, clinician perspectives, patient perspectives, and economic factors, with the ultimate goal of making a non-binding reimbursement recommendation to the provinces and territories of Canada, except Quebec [17]. A similar process is undertaken via Quebec’s INESSS, although the approach is modified to consider “societal factors” [18]. Patient and physician groups have an opportunity to provide input, and, in Quebec, individual citizens can also submit their feedback to INESSS.

The pCODR Expert Review Committee (pERC) makes the final funding recommendation for all cancer drugs that are reviewed through the CADTH process by following a defined deliberative framework [19,20]. Appeals are allowed following the draft recommendation before the final recommendation is issued. No appeal is allowed for Quebec’s INESSS reviews (although that province features a more generous and separately adjudicated individual patient review program, which may provide access to medicines that are rejected by INESSS).

CADTH has published target timelines for its process, which is expected to take approximately 200 days from the time of screening/initiation of the review until final recommendation [21]. Submissions are reviewed on a first-come, first-served basis [19]. CADTH comes close to meeting its timeline target as it takes, on average, 216 to complete a review of a cancer medicine [22]. Previous data have suggested that the pCODR HTA took longer, with a median time of 8.7 months among 21 drugs reviewed from 2011 to 2016 [23].

CADTH consistently states that it considers evidence from one or more randomized controlled trials (RCTs) to be the preferred form of clinical evidence, but it does consider data from non-randomized studies when an RCT is impractical when RCTs have limited external validity when evaluation of important clinical events requires longer-term follow-up, and in several other scenarios [21]. For many years it would seem that comparative data demonstrating clear overall survival (OS) and progression-free survival (PFS) outcomes were necessary in order to receive a positive recommendation. For example, pCODR recommended not to fund dabrafenib/trametinib in the treatment of mutated BRAFV600 NSCLC in 2017 because of the absence of comparative data for OS and PFS [24].

However, more recently, a number of treatments in rare lung cancer subtypes received a positive HTA recommendation based on single-arm phase 2 studies where randomized data were unattainable, including a new successful submission for the previously rejected dabrafenib/trametinib combination in mutated BRAFV600 NSCLC [25] and critozinib in ROS1 mutated lung cancer [26]. 

It is important to note that the HTA agencies are owned, funded, and managed by the governments to whom they provide recommendations and receive submission fees from drug developers. These governance and funding arrangements may give rise to conflicts of interest.

### 1.5. Pan-Canadian Pharmaceutical Alliance

The pCPA is the national price negotiator for drugs sold in Canada, with the goal of obtaining the greatest value from recommended treatments for publicly funded drug plans. pCPA participants include all provincial, territorial, and some federal drug plans. It was first established in 2010 by the Council of Federation (a collective of provincial and territorial premiers). 

The pCPA became a more formalized entity in 2015 and was joined by Quebec and the federal drug plans by 2016 [27,28]. It claims to have saved $2.9 billion dollars in annualized savings from its negotiated deals as of March 2021 [28].

Price negotiations for new drugs take place after CADTH (or INESSS in Quebec) issues a final recommendation. The decision to undertake negotiations on a given drug is based on criteria gleaned from the HTA recommendation and other factors [28]. If the participating plans decide they want to negotiate, one of the participating jurisdictions or the pCPA office (staffed by Ontario government officials) takes the lead in the negotiation. In most cases, pCPA negotiations involve all jurisdictions, although individual drug programs can opt-out. 

The pCPA has been criticized for a lack of transparency [29], with little publicly available information on their framework and governance, and without clear parliamentary oversight. While the negotiated prices of generic medications are transparent and generally apply to the whole market, the negotiated prices of innovative medicines are confidential and only apply to the participating payers. Further, the pCPA does not publish formal review or negotiation timelines, nor the criteria used in decision-making [30]. Studies have suggested that the time from the CADTH pCODR recommendation to the pCPA negotiation publication for oncology drugs took as long as 371 days in 2016 [30]. 

### 1.6. Provinces and Territories

Once the HTA has been finalized and the price of the drug is agreed upon, it remains at the discretion of each provincial and territorial payer to decide whether and when a given drug will be funded, within the context of its own budget and formulary. Each drug plan may make its own reimbursement decision based on the HTA agency’s non-binding recommendation, the LOI terms negotiated by the pCPA, as well as the plan’s mandate, priorities, and financial resources [17]. Provinces may also initiate a separate and additional HTA review of new medicine to inform their funding decision. In this context, individual jurisdictions may introduce additional or unique funding criteria and coverage limitations [29], may fund at different time points or may choose not to fund a given drug at all. As such, there may be considerable variability in access across Canada based on the province or territory of residence. 

There is a wide range of timelines between LOI and funding decisions across provinces. It has been shown that for a selection of 15 cancer drugs funded in at least two provinces between 2011 and 2016, the time intervals from first to last provincial funding ranged from 2.8 to 23.8 months [23]. For example, in the treatment of metastatic breast cancer, pertuzumab was publicly funded in British Columbia in November 2013 but did not receive public funding in Prince Edward Island until about 1.5 years later, in April 2015 [31]. Such inequity of access has been the basis of some of the arguments for national pharmacare [32]. 

One reason for the variability could be differences in how clinicians and patients access funded treatment across the provinces. The three westernmost provinces—British Columbia, Alberta, and Saskatchewan—fund and administer all oncologic drugs and cancer-related therapies, both oral and intravenous compounds. Manitoba and Ontario also have cancer agencies; however, funding decisions and administration can vary depending on the type of cancer drug prescribed. Oral therapies in Ontario and Eastern Canada are dispensed by community pharmacies and paid for through the provincial public drug programs, while intravenous oncologic drugs are administered in hospitals and paid for by the cancer programs. Quebec’s system relies on individual hospital budgets for intravenous oncologic drugs, and the institutions are reimbursed for those expenses from the province’s health ministry.

To add to the variability across the country, there are some federal drug plans that may provide funding to specific subsets of the population regardless of province of residence (i.e., the Non-Insured Health Benefits Plan for Indigenous Canadians, the Canadian Forces Drug Benefit Plan, Veterans Affairs Canada Treatment Benefits Program, among others).

### 1.7. Other Mechanisms of Access

The process outlined thus far describes the conventional, if complicated, path for approval and public funding of a cancer drug.

While the drug is working through that process, there may be alternate options for access:

1. As stated earlier, if NOC has not yet been granted, but clinical evidence exists for efficacy of a drug, clinicians can apply to HC’s Special Access Program (SAP). This does not provide funding for the drug, but some pharmaceutical companies will provide compassionate access following SAP approval;
2. After NOC, but before public funding, drugs can be funded through private insurance or individual private pay (one author has had a patient use ^®^gofundme to raise resources for a $10,000 CAD per month medication). These options are sometimes supported by manufacturer-funded compassionate programs that will support co-pay costs, or on occasion provide free access. Other companies may provide a rapid ‘bridging access’ program where free drugs are supplied while other mechanisms of funding are being sought;
3. Clinical trials can also provide access to treatment, and some companies may open trials with loose inclusion criteria to allow more patients to receive the drug. “Phase 4” studies, which are initiated after a drug has already received NOC, may also provide access to medicines. They are also referred to as “post-marketing surveillance” studies, where the investigators can learn more about the long-term use of a drug;
4. Hospital budgets, in some jurisdictions, are a source of access to some medications; however, this is rarely a sustainable solution given institutional budget constraints. In Quebec, however, cancer treatment funding is managed through hospital budgets;
5. Provincial programs may allow for “special authorization” through exceptional access program applications. These differ from province to province.

## 2. Discussion

The Canadian system for approval, evaluation, and funding of new drugs is complicated. The multi-step process characterized by different agencies, lack of transparency, and apparent duplication of effort, can lead to long delays from the time of proven drug efficacy to its routine availability. Even after successfully working through HC, CADTH, and pCPA, there are discrepancies in timely access based on province or territory.

In 2020, the Canadian Partnership Against Cancer (CPAC) published a report entitled “Lung Cancer and Equity” [33]. While this report does not specifically highlight drug access, it does shine a light on challenges in lung cancer care with respect to Canada’s rural, poor, and Indigenous populations. Equity is a problem in the healthcare system, and this is also reflected in the approval processes.

Ultimately, Canadians usually do get access to effective cancer drugs, yet the multi-step process allows us to identify opportunities for improvement.

### 2.1. Duplication of Effort

Some considerations might include reviewing why both PMPRB and pCPA are involved in setting drug prices. If PMPRB regulates lower ceiling prices of cancer drugs, presumably the pCPA would not be needed to negotiate a lower price at all. Conversely, if cancer medicine prices, which are primarily funded through public payers, can be negotiated by the pCPA, what is the de facto role of the PMPRB?

Regarding Quebec, while the HC and pCPA processes cover all provinces and territories, there is duplication in effort with both CADTH and INESSS performing HTA reviews, although there are alignment and resource-sharing initiatives underway among the agencies. 

### 2.2. Timeliness

While HC and CADTH work to defined target timelines, and the processes can work in parallel (although CADTH does not make a recommendation until NOC is granted), there is no such timeline for the pCPA. Are there opportunities to mandate conditional funding agreements following a positive CADTH/INESSS review, to allow market access while the final price is being negotiated? These conditional funding agreements are seen in other countries such as Germany and the United Kingdom and would negate the often year-long “black hole” awaiting public funding for an HTA-approved drug.

### 2.3. Efficiency

Outcomes based agreements (OBA) are a potential approach to increase spending efficiency where an agreement between manufacturer and payer can see refunds or rebates to the payer based on the performance of the drug in the market, acquired from real world evidence and pre-defined metrics. Alternatively, individual OBAs have been postulated to ensure that public payers reimburse only effective treatments, whereby the manufacturer provides free drugs to a given patient for a short period until efficacy can be ascertained, at which point the public payer assumes funding.

Lastly, the approval and funding process includes no mechanism to de-list previously funded drugs that may no longer be considered as clinically relevant, with diminishing value as cancer treatment evolves. Considering mechanisms to de-list previously publicly funded drugs may be an avenue to increasingly fund newer, more effective drugs in a timely manner.

## 3. Conclusions

In conclusion, this paper outlines the multi-step process for the approval of new cancer drugs in Canada. The reader should have a clearer understanding of the complexities of this process and some of the current debates and evolution in various components.
